# Psychophysical evidence for a non-linear representation of facial identity

**DOI:** 10.1016/j.visres.2009.06.016

**Published:** 2009-09-09

**Authors:** Steven C. Dakin, Diana Omigie

**Affiliations:** a UCL Institute of Ophthalmology, University College London, EC1V 9EL London, UK; b Department of Psychology, Goldsmiths, University of London, SE14 6NW London, UK

**Keywords:** Face perception, Discrimination, Identity, Dipper function, Average face

## Abstract

It has been proposed that faces are represented in the visual brain as points within a multi-dimensional “face space”, with the average at its origin. We adapted a psychophysical procedure that measures non-linearities in contrast transduction (by measuring discrimination around different reference/pedestal levels of contrast) to examine the encoding of facial-identity within such a notional space. Specifically we had subjects perform identity discrimination at various pedestal levels of identity (varying from average/0% to caricature/125% identity) to derive “identity dipper functions”. Results indicate that subjects are generally best at spotting identity change in neither average nor full-identity faces, but rather in faces containing an intermediate level of identity (which varies from face-to-face). The overall pattern of results is consistent with the neural encoding of faces involving a single modest non-linear transformation of identity that is consistent across faces and subjects, but that it scaled according to the *distinctiveness* of the face.

## 1 Introduction

Recently, progress has been made in understanding how humans encode facial-identity, using the concept of *face space*, where each face is represented as a point within a multi-dimensional space centred on the average face (e.g. Valentine, 1991), and where more distinctive faces fall further from the average/centre. Conceptually, this framework can, for example, explain people’s difficulty in differentiating between members of ethnic groups other than their own (Lindsay, Jack, et al., 1991) based on the notion that own-race faces occupy high-density regions around the average while other-race faces lie within a cluster some distance away (Valentine, 1991). Similarly, arguing that distinctiveness is related to a face’s distance from the mean has been used to explain our superior recognition of distinctive compared to more typical unfamiliar faces (Hancock, Burton, et al., 1996).

**Fig. 1:**
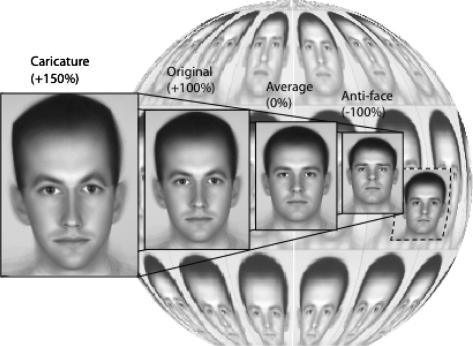
A schematic representation of face space (Leopold, O’Toole, et al., 2001). Identities are coded relative to an average in the centre. Faces falling on a single “identity trajectory“ are framed in solid black. As one moves along the ID trajectory, distinctiveness decreases, progressing from caricatures through the original, to the (minimally distinctive) average. Passing beyond the average along the same ID trajectory leads to anti-faces that may be similar to real faces (as indicated by the example of the real face in a dashed framed).

With respect to the neural encoding of identity, a key concept is the *identity trajectory* (Fig. 1), a trajectory in face space passing through an individual face and the average. Faces on this trajectory each contain some percentage of the face’s identity. If it is greater than 100% the face is a caricature, which exaggerates differences between the individual and the average. Such faces are identified more accurately and quickly than the original face (Lee, Byatt, et al., 2000; Rhodes, Brennan, et al., 1987). Conversely *anti-faces* – which fall on the identity trajectory on the opposite side of the average face – contain a negative percentage of identity and are effectively “opposites” of the original. The main evidence for the psychological importance of the identity axes comes from psychophysical studies of adaptation. It is known that prolonged exposure to an adapting face shifts the perceived facial categories of a subsequently viewed (test) face away from the adaptor, e.g. prolonged viewing of a happy face makes a subsequently viewed emotionally-neutral face appear sad (Webster, Kaping, et al., 2004). In the case of identity, adaptation to an anti-face shifts the appearance of an average test-face towards the identity of the original (Leopold, O’Toole, et al., 2001), and the magnitude of such adaptation is greater for anti-faces than for faces that are equally dissimilar to the test (Rhodes & Jeffery, 2006). Furthermore, effects transfer (at least partially) when the appearance of the test has been transformed via changes in expression (Benton & Burgess, 2008), although there is weaker transfer when the adapting and test-faces have different poses (Benton, Jennings, et al., 2006; Jeffery, Rhodes, et al., 2006) or orientations (e.g., if the adapter is rotated compared to the test (Watson & Clifford, 2006). This would seem to indicate that adaptation, at least in part, taps into a higher-level facial-coding mechanism.

Loffler, Yourganov, et al. (2005) used fMRI to examine if the coding strategy employed by neurons in the human brain operates relative to some average/norm. The BOLD signal from the fusiform face area (Kanwisher, McDermott, et al., 1997) increased with the degree of deviation from average for faces falling along a single identity trajectory. Furthermore, presenting faces that deviated along axes that were *orthogonal* to the identity axis did not lead to a comparable increase in BOLD response. Leopold, Bondar, et al. (2006) measured response of single neurons in the macaque inferotemporal (IT) cortex in response to a range of photographic facial stimuli spanning nine points in an identity trajectory from average (0% identity), to an individual’s face (100% identity), to a caricature (160% identity). Neurons exhibited a marked tendency to show tuning centred on the average face and for their firing rates to increase near-linearly with increasing identity-strength. These findings are broadly consistent with an average-based encoding of facial identity.

**Fig. 2:**
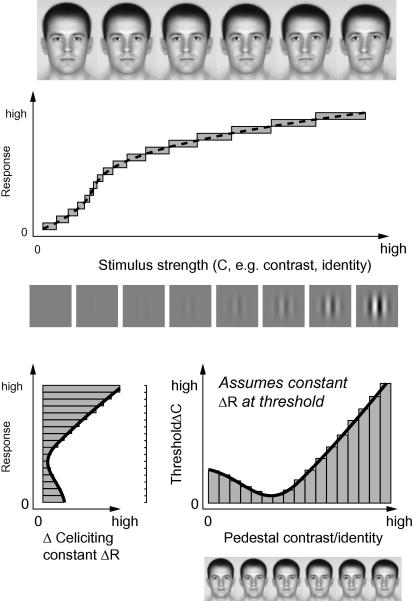
Logic of the approach. Notional neural response (dashed line) elicited by different stimulus strengths (either contrast or facial identity). The width of the bars indicate the amount of extra stimulus strength required to elicit a constant change in response (bar-height) at different points along the response function. Bars are collapsed in the lower left graph to highlight that, for this response function, there is an optimal baseline (*pedestal*) stimulus strength (falling on the steepest part of the response function) that requires only a small change in stimulus strength to elicit this response change. Plotting the change in stimulus strength supporting a constant response change, as a function of baseline stimulus strength, gives the profile shown in the bottom right. Assuming that discrimination requires a constant change in response, the solid black line is the predicted change in stimulus strength supporting a just-noticable difference at differing baseline levels of stimulus strength. By psychophysically estimating the minimum additional stimulus strength that can be reliably noticed by observers, at different baseline (pedestal) levels of stimulus strength, one can essentially integrate the resulting data to infer the underlying response function.

Two studies examining how distance from the average face affects subjects’ ability to discriminate between faces, have produced contradictory results. Wilson, Loffler, et al. (2002) report that discrimination between morphed faces is more difficult when they are further away from the mean face. This could reflect chronic adaptation to near-average faces (which might improve sensitivity) an idea Rhodes, Maloney, et al. (2007) tested using three tasks probing face processing either near or far from the average. Subjects’ ability to judge inter-ocular spacing was unchanged with distance from the mean, whereas perceived similarity (assessed using ratings) and perceptual difference scaling both indicated *reduced* sensitivity to facial difference for near-average faces. We sought to explore this issue in detail by using behavioural psychophysics to examine the nature of neural encoding of faces along the identity axis. Our specific aim is to infer the full underlying *identity response functions* (IRFs) that support discrimination of faces differing in identity-level. To do this we rely on previous paradigms that use psychophysically measured thresholds to infer underlying response functions for stimulus *contrast* (Zenger-Landolt & Heeger, 2003). Specifically we used a *pedestal paradigm*, measuring how much extra identity had to be added to a face for it to be just discriminable from two reference/distracter faces (which are presented at a fixed pedestal identity level). We also sought to compare discrimination for inverted and polarity-inverted faces (which are matched to the upright stimuli in terms of the physical distortion applied.) The advantage of a pedestal paradigm is that it provides a full mapping of discriminability as a function of identity-level allowing one to infer the underlying response function that supports such discrimination, as illustrated in Fig. 2 (for a recent review of this paradigm see Solomon (2009)).

## 2 General methods

### 2.1 Stimuli

**Fig. 3:**
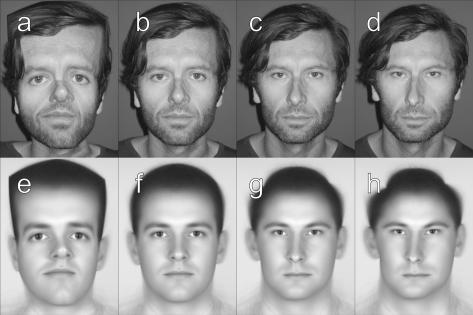
(a–d) Photographic stimuli morphed into registration with key-points consistent with a varying amount of identity: (a) −150%, (b) 0%, (c) 100%, (d) +150%, (e–h) same but showing morphed versions of the *average face* (f).

We obtained full-face uniformly-lit photographs of 64 male subjects (e.g. Fig. 3c) and manually located 32 key-points (around the eyes, nose, etc.) using digital versions of these images. We co-registered all faces by first averaging all key-point sets and then morphing each face into registration with this averaged set (e.g. Fig. 3b). Morphing was done using custom software written in the MatLab programming environment (MathWorks) relying on built-in 2D bilinear interpolation routines (interp2) to perform image stretching. Averaging the resulting 64 morphed images gives the “average face” (Fig. 3f). All our stimuli were constructed by morphing this average face into registration with different sets of key-points. Morphing the average back to the original set of key-points for a given face gives a 100% identity stimulus (Fig. 3g). We could also generate faces that had various identity “strengths” by morphing the average into registration with an arbitrary positive or negative proportion of the difference between the average key-points and the individual key-points:

(1) $$x_{\text{morph}}=x_{\text{average}}-\frac{W}{100}(x_{\text{average}}-x_{\text{face}})$$

Here $x_{\text{face}}$ represents key-points from an individual face, and $x_{\text{average}}$ the key-points from the average.

*W* is the identity strength, where for example, −100% represents the anti-face, 0% the average, 100% the original identity and 150% a caricature. Examples of such stimuli are shown in Fig. 3e–h. Using morphed averages rather than morphed originals has the advantage that all faces are matched for colouration and texture, (which cannot be easily parameterised) so that the only differences between stimuli are attributable to changes in face geometry due to the morphing.

### 2.2 Equipment

Stimuli were presented on a Lacie CRT monitor attached to an Apple iMac computer. The display had 1280 × 960 pixel resolution and operated at a refresh rate of 75 Hz. The monitor was viewed at a distance of 1 m. Subjects responded using a numeric keypad.

### 2.3 Subjects

*Rating experiment*: Subjects were 12 members of staff at the UCL Institute of Ophthalmology with normal or corrected-to-normal vision, unfamiliar with the face-set.

*Detection and discrimination experiments*: In the discrimination experiments the two authors (SCD and DEO) served as subjects along with two naïve subjects (JRC and DAK). Three of our subjects were male Caucasians, including two (SCD and DAK) native UK residents, and one (JRC) Australian who had been resident in the UK for a year at the time of testing. The fourth subject (DEO) was a female of mixed (Nigerian-English) ethnic origin resident in the UK for 8 years prior to testing. Thus all of our subjects would have been exposed continuously to specifically British Caucasian faces for at least a year prior to testing. Subjects in both experiments had normal or corrected-to-normal vision acuity. All were experienced at participating in psychophysical experiments.

### 2.4 Procedure

#### 2.4.1 Rating experiment

**Fig. 4:**
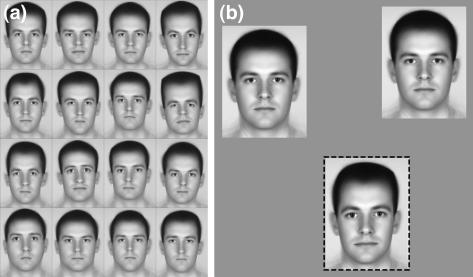
A typical trial from (a) the rating and (b) the detection experiment. (b) The odd-man-out (dashed frame) has an identity level of 75%; the other two faces are averages (identity level 0%).

Random subsets of 16 out of the 64 possible greyscale (morphed average) face images were presented on the monitor in a 4 × 4 grid (Fig. 4a). Each face image was 300 × 200 pixels so the 4 × 4 grid subtended 7.4° (width) by 9.8° (height). Subjects were presented with a randomly selected face-grid displayed on a grey background and required to indicate “Which face is the most distinctive?” by clicking on it using the mouse. No time limit was given and reaction times were not recorded. Once clicked, faces were occluded with a grey block, and the subjects made their judgment using the remaining faces until only one face remained. Subjects performed this task on a total of eight grids each containing a new subset of 16 faces. If a face was clicked first it achieved a score of 16, if it was clicked second, it achieved a score of 15 and so on. Ratings were compiled by averaging each face’s score across presentations and subjects.

#### 2.4.2 Discrimination experiments

The first time a given test-face was used within any experimental session subjects studied the 100% identity *test-face* for at least two minutes, in order to familiarise themselves with it, prior to commencement of testing. These test-faces were selected to span the space of rated distinctiveness. Experimental runs consisted of 64 trials. On each trial a triplet of faces was presented; two reference faces (baseline stimuli) and a target-face. The three faces were randomly positioned on an annulus such that they maintained a constant distance from central fixation (4.2°) and from one another. Faces appeared simultaneously on a uniform grey field for a duration of 500 ms (Fig. 4b). Each face subtended 3.7° (width) by 4.9° (height). After 500 ms – an exposure duration selected to avoid subjects being able to scrutinise local differences between faces – all faces were replaced with boxes labelled “1”, “2” and “3” where they had stood. Observers were instructed to indicate “which face was the odd-man-out?” by hitting keys “1”, “2” or “3”. There was no time limit for responses but observers rarely took more than 2–3 s to respond. The observers’ response initiated the next trial. Observers were aware that two faces were always identical (the reference faces) and one always different (the target).

**Fig. 5:**
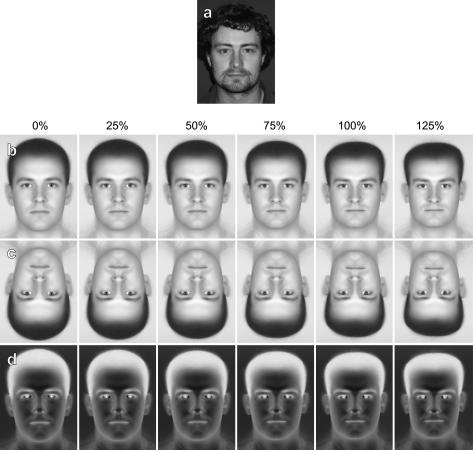
(a) Original image. (b–d) Examples of stimuli derived from this face, ranging from an average (0% identity strength) to a caricature (125% identity). Images shown are from (b) upright, (c), inverted and (d) contrast-polarity inverted conditions. Note how much harder it is to discriminate changes in the stimuli shown in (c) and (d), compared to (b).

The target was made of the average face with some proportion of the test-face identity added to it. The amount of identity contained in the target was adjusted from trial-to-trial using an adaptive staircase (Watson & Pelli, 1983) to determine the *threshold identity level* (i.e. one supporting 82% correct performance). After detection thresholds were measured for a subset of about 12 faces, four were selected that spanned the range of rated distinctiveness and physical distance from the average, while at the same time having detection thresholds of varying magnitude. These faces were then presented not just as averages but with some (variable) level of “pedestal” identity added to them. Pedestal identity was varied (across runs) from 0% to 125% in steps of 12.5%. Subjects indicated the odd-man-out using the detection procedure. Runs were performed (in random-order) using: (a) upright/positive-polarity faces, (b) inverted/positive-polarity faces and (c) upright/negative-polarity faces. Examples of these stimuli are shown in Fig. 5.

## 3 Psychophysical estimation of the identity response function

**Fig. 6:**
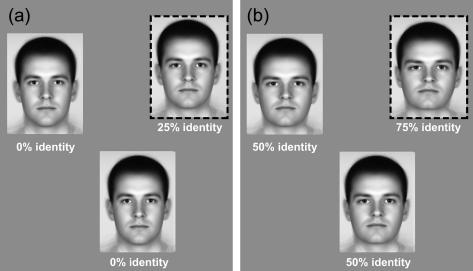
Examples of two typical discrimination trials. (a) Two identical reference faces which contained a ‘‘pedestal‘‘ level of identity (here 0%), and one target-face (dashed frame) whose identity-level was the pedestal plus some increment (0% + 25% = 25%). Subjects reported the ‘‘odd-man-out‘‘ (indicating the numeric label that replaced each face after 500 ms). (b) Is similar except that now the two references contained a pedestal identity level of 50% and the target (dashed frame) contained 50% + 25% = 75% identity. Although in both halves of the figure the target-face is defined by a 25% increase in identity (compared to the reference faces) it may be easier to spot the odd-manout in (b); the pedestal improves subjects‘ ability to spot an identity-increment. This is a signature of a ‘‘dipper. discrimination function shown in the lower right section of Fig. 2, i.e. of a non-linear representation of identity.

**Fig. 7:**
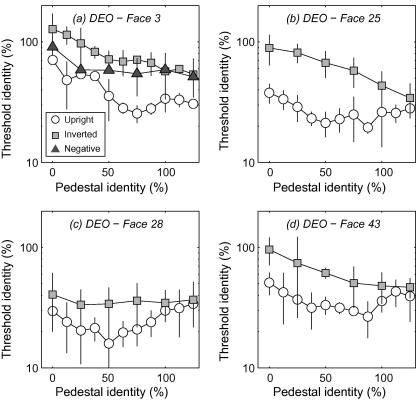
Threshold-versus-identity (TvI) curves for subject DEO: the minimum amount of additional identity supporting odd-man-out discrimination as a function of pedestal identity level, for four different faces. Thresholds are uniformly lower (indicating better performance) for upright, positive contrast-polarity faces compared to the other conditions and generally drop with increasing pedestal identity. Critically, TvI functions with upright positive-polarity faces shows a shallow “dipper” type pattern, consistent with non-linear representation of identity.

**Fig. 8:**
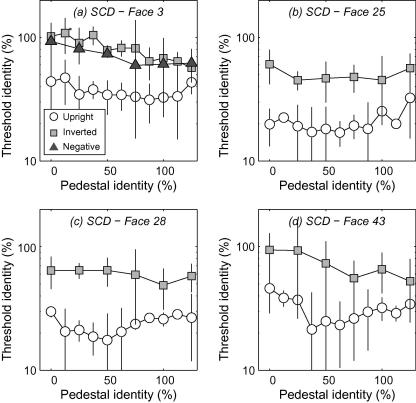
As Fig. 7 for subject SCD.

**Fig. 9:**
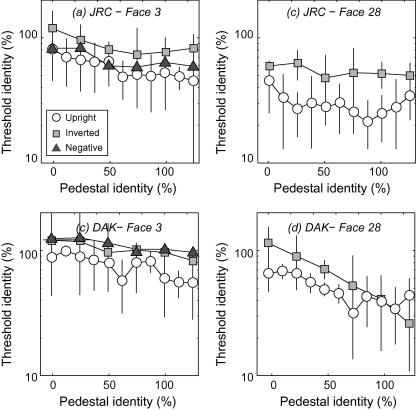
As Fig. 7 for two naïve subjects’ performance with faces #3 and #28.

We measured the discriminability of faces falling on a given identity axis, as they varied in identity-strength; examples of two experimental trials, with corresponding pedestal levels of 0% and 50%, are shown in Fig. 6a,b. Both of the odd-men-out (dashed frames) have 25% more identity than the distractor faces which have identity levels of (left) 0% and (right) 50%. It is easier to spot the odd-man-out on the right when the “pedestal” identity levels of the distractors are higher. This tendency is borne out in the full threshold-versus-identity (TvI) curves shown in Figs. 7–9.

TvI curves from the three presentation conditions (upright, inverted and polarity-inverted) and the four faces (averaged across 3–6 runs) are presented for three observers in Figs. 7–9. Subjects were generally better at identifying the target (i.e. thresholds were lower indicating less additional identity was required) as the reference faces had more identity introduced into them. This is consistent with earlier work using faces morphed along the identity axis (Rhodes et al., 2007) However, for the upright face conditions, data showed a shallow “dipper” shape: at low identity levels thresholds initially drop with increasing pedestal identity, but then steadily rise at higher identity pedestals. In terms of an underlying identity response function (IRF) these findings are consistent with a non-linear transduction of identity level. Dips were particularly evident for face #28 (in all subjects), face #43 (for experienced observers) but were generally much weaker for face #3 (for all subjects). Below we explore the possibility that these differences are attributable to differences in the distinctiveness of faces. By contrast, performance with inverted and contrast-polarity inverted faces was generally poorer at low pedestal identity levels (i.e. for tasks close to standard detection) and exhibited straightforward improvement with increasing identity level (i.e. inverse Weber’s law behaviour) with no dips. The overall pattern of results led to discrimination around high pedestal identity levels being almost identical across upright and inverted/polarity-inverted faces. This is a rather counter-intuitive finding we return to in the Discussion. The monotonic trend in thresholds we observe in inverted/polarity-inverted conditions is important because such stimuli contain similar physical distortions to the upright face condition. Our not finding dips with inverted/polarity-inverted stimuli demonstrates that non-linear discrimination performance is related to face perception and not to, e.g. subjects general ability to detect changes in image distortion.

## 4 Modelling the identity response function (IRF)

**Fig. 10:**
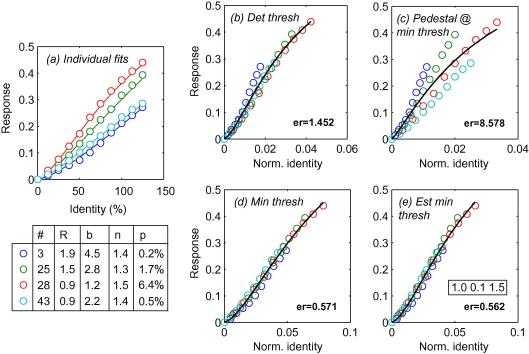
(a) Circles show empirically derived (data from DEO) estimates of the identity response function for four faces, along with fits from the Naka–Rushton (NR) equation (solid lines). The boxed legend below the figure indicates the best fitting parameters for each individual NR fit to the data (*R*_max_, *b*, and *n*); note the shallow sigmoidal shape of the response function as indicated by values of *n* consistently exceeding 1. The right part of the figure shows these response function re-plotted on *x*-axes that have been scaled by one of the four parameters indicated. Scaling by the estimated minimum threshold brings the curves into close registration.

**Fig. 11:**
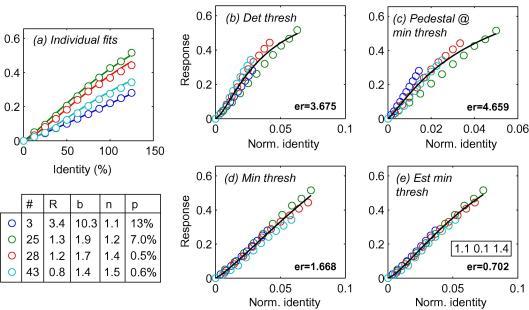
As Fig. 10 for subject SCD.

Identity response functions (IRFs) were obtained by integrating the psychophysical threshold versus identity (TvI) curves. First the thresholds were converted to sensitivity values and were cumulatively summed. Results were then fit with a Naka–Rushton function:

(2) $$R=R_{\text{max}}\frac{x^{n}}{\left(b^{n}+x^{n}\right)}$$

using the fmins function in Matlab. Within this expression the parameter $R_{\text{max}}$ scales the overall response, *b* sets the semi-saturation point, and *n* set the degree of non-linearity of the response function. Figs. 10 and 11 show response functions derived from the threshold versus identity curves for experienced observers DEO and SCD, respectively. Note that the response functions obtained, showed a slight non-linearity (*n* = 1.1–1.5) as a result of the dips in TvI functions. We next used a bootstrapping procedure to determine the statistical significance of these dips. For each observer we used the mean and variance of the thresholds obtained at each identity level to generate random threshold distributions. We then generated a new data set by drawing one sample from each of these random distributions at each identity level and repeated this procedure to generate 1024 artificial data sets. We transformed each of these data sets into cumulative sensitivity values (in the manner described above), fit them with a Naka–Rushton function and recorded the distributions of fit values obtained over the 1024 sets. The percentage of values of *n* falling below 1 (i.e. indicating a fit that was inconsistent with a dip) gives a measure of statistical significance of the dips (*p*). These estimates are tabulated below Figs. 10a and 11a for subjects DEO and SCD, respectively. Dips were statistically significant in 8/12 conditions tested. Specifically they were significant in all but one case for DEO (when *p* approached significance at 6.4%). For SCD the dip was not significantly significant for face 3, approached significance for face 25 (*p* = 7%) and was highly significant for remaining faces. For the naïve subjects, each tested on faces 3 and 28, *p* was 2.5% and 7.5% (JRC) and 1% and 0.5% (DAK), respectively.

We wondered if differences between IRFs (across conditions and subjects) could be accounted for by scaling of a single underlying IRF. Scaling simply consists of re-plotting response functions on a new *x*-axis that is a scaled version of the identity pedestal-level. Figs. 10 and 11 also show the IRFs from both experienced subjects’ for the four faces, re-plotted using *x*-axes that have been normalised using four different values:

(1) The detection threshold for each face (i.e. the threshold with a 0% pedestal).
(2) The pedestal value that induced the minimum discrimination threshold.
(3) The minimum discrimination threshold (obtained experimentally).
(4) The estimated minimum discrimination threshold. (This is estimated by using the best fitting response function, taking its inverse and derivative to predict the TvI, and then estimating the minimum threshold that would be obtained with the optimal pedestal. This is similar to (3) but uses all the experimental data and is therefore less prone to sampling limitations).

From Figs 10 and 11 it is evident first that rescaling by detection threshold for each face does a poor job of co-registering the response functions, calling into question the role of the average face as a natural “origin” for our discrimination data. However, rescaling the *x*-axis using the estimated minimum threshold brings the response functions from the four faces into close registration, for all subjects. Error values were consistently lowest using this scheme. Note also the similarity of the estimates of the three parameters of the best fitting single IRF to both subjects (1.0, 1.1, and 1.5 for DEO versus 1.0, 1.1, 1.4 for SCD) suggesting that the underlying IRF may be common across subjects as well as across faces.

**Fig. 12:**
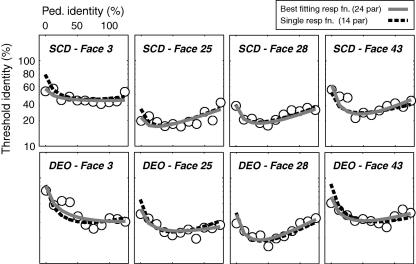
Graph showing thresholds obtained for the upright faces with line of best fit. (grey line) The broken line shows the derivative of the Naka–Ruston function of our model response function obtained by rescaling the *x*-axis of all four faces to their estimated minimum threshold.

These results indicate that subjects’ performance on the pedestal experiment were consistent with a modest non-linear transduction of stimulus identity. In addition our data are consistent with a single underlying identity response function that was simply scaled by the estimated lowest discrimination threshold for an individual face. To further validate our use of a generic scaled IRF, we used it to go back and predict the original thresholds (TvI function) for each subject. By taking the derivative of the scaled (using estimated minimum threshold) best fitting scaled IRF for each subject, and inverting it, we were able to generate such predicted thresholds. Fig. 12 shows the result of doing this. For subjects SCD and DEO, the line of best fit (solid grey line) and the predicted TvI function (broken black line) show substantial agreement. This is impressive given that the fits for each subject now use only 7 parameters (3 for the generic response function +4 scaling parameters for each face) compared to the original 12 (4 × 3-parameter independent fits). Arguably four of the seven parameters in the generic model fits are not free since they were derived empirically.

## 5 The role of distinctiveness in determining the identity response function

**Fig. 13:**
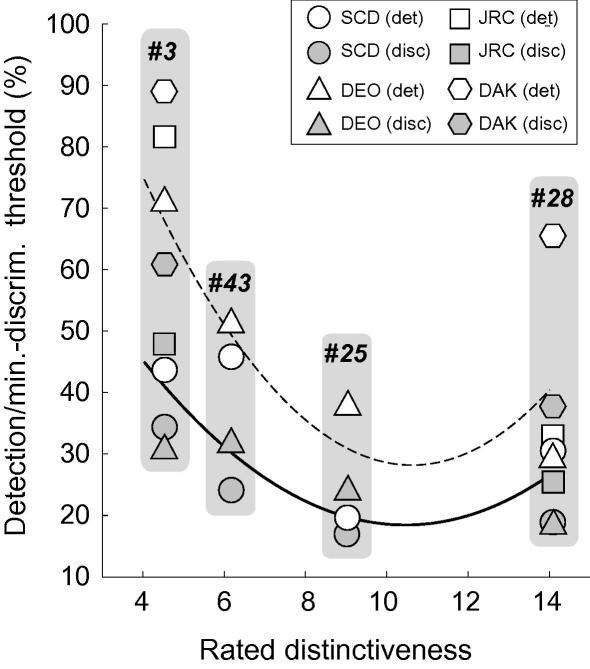
Plots of rated distinctiveness against (open symbols) identity detection thresholds and (filled symbols) estimated minimum discrimination thresholds, for the four face images used in this study. Note that distinctiveness appears to be more closely related to the estimated minimum discrimination thresholds than the detection thresholds.

The last section demonstrated that identity response functions appear to be scaled by the minimum detectable change in identity for a given face. What property of faces sets this value? We next asked if this property might be related to facial distinctiveness by relating our earlier results to distinctiveness ratings for all the faces used so far. Fig. 13 plots detection thresholds and estimated minimum discrimination thresholds (EM; from Section 4) against rated distinctiveness. Note that both detection thresholds and EMs generally fall with increasing distinctiveness. A second-order polynomial fit to these data (solid and dashed lines in Fig. 10) account for 50% and 29% of the variance in the discrimination and detection data, respectively. A more reliable trend is evident in the relationship between EM and rated distinctiveness.

## 6 Discussion

To summarise, we have shown three things:

(1) Discrimination based on identity strength improves (for upright and inverted faces) as the stimuli move away from the average.
(2) Best discrimination of upright faces occurs around intermediate levels of identity, consistent with a modestly non-linear transduction of identity.
(3) Results are consistent with a single underlying identity response function that is scaled by a parameter related to distinctiveness.

### 6.1 Morphing and image distortion

When faces are morphed, one essentially imposes a form of image distortion or disorder. In general our ability to detect disorder shows a Weber’s law dependence (i.e. becomes more difficult) as a function of increasing image-disorder (e.g. Levi, Klein, et al., 2000), but this is typically assessed using arrays of regularly spaced elements all of which undergo disruption from the same probability density function. We propose that the finding that our ability to detect facial-image distortion increases with increasing pedestal distortion (point 1) likely arises from subjects increasingly being able identify the *local-features* undergoing the largest distortions (since the distortion applied under morphing will be highly non-uniform across the face). As pedestal distortion increases, all images appear “strange”, allowing one to identify the *most distorted* features in those images, and so to make an essentially local-feature comparison amongst stimuli. This would explain a counter-intuitive outcome of the first experiment – that thresholds for inverted and upright faces could converge at high identity levels. Stimuli morphed beyond the 150% identity level (e.g. Fig. 3d) quickly stop resembling physically realistic faces, so that subjects will rely less and less on strategies related to face perception (which are decreasingly appropriate at identity levels beyond 100%) and switch to trying to spot gross physical distortion of the stimulus. Such strategies must be more related to local structure (since subjects would be unlikely to have previously encountered the particular combination of distorted features presented) i.e. will depend less on the holistic structure of faces that is thought to confer the advantage on upright faces. A move to increasingly local processing would also explain why IRFs for inverted faces are shallower i.e. show less dependence on image distortion; inverted faces are thought to be processed in a less holistic/more local manner to start with (Goffaux & Rossion, 2006; Rossion, 2008; Yin, 1969).

Consistent with the view that effects of identity strength are contingent on tasks tapping into global/holistic processing, Rhodes et al. (2007) failed to find an effect of identity-strength on subjects ability to judge eye-separation (an essentially local judgement). Later experiments in the same paper did use more global tasks (similarity ratings and perceptual difference scaling) and reported that processing was generally poorer around the average. This closely accords with our overall finding (point 1) of an inverse Weber’s law dependence on identity strength.

**Fig. 14:**
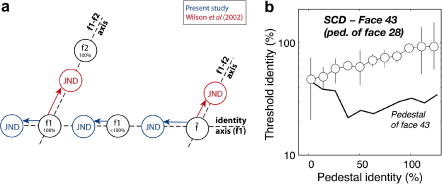
(a) Schematic face space showing the identity axis used to generate stimuli in this study along with the axes employed by Wilson et al. (2002). Dark grey and light grey discs indicate faces with a just-noticeable-difference (JND) in identity for this and the Wilson et al. study, respectively. Note the very small JND at an intermediate level of identity for this study, and that JNDs are greater at higher levels of identity in the Wilson et al. study. Because different projections were used to generate stimuli in both studies, their findings are not mutually inconsistent. (b) Results from a control experiment conducted to examine if dips in TvI functions depended on having the target-face as a pedestal. The solid line shows the non-linear TvI function derived in the earlier experiment, and the open symbols shows data collected using stimuli that were essentially identical except that the pedestal-face was now not matched to the target. As the identity level of the mismatched pedestal-face increases we observe a steady increase in masking (with no dips).

Contrary to this position, Wilson et al. (2002) has shown that faces further from the mean are *more difficult* to discriminate from one another than typical faces. Using *face cubes* – face subspaces comprised of four faces made mutually orthogonal with respect to any given face – Wilson et al. showed that discrimination thresholds are about 1.45 times larger for face cubes centred away from the mean than from those centred at the mean. The authors reasoned that if most faces we experience are similar to the mean, then it is most necessary to be able to discriminate among this population and thus lower thresholds would be observed near to the mean than further away from the mean. By contrast, our findings showed improved discrimination of faces with increasing distance from the mean. However, while Wilson et al. measured discrimination between faces of different identities, our experiments looked at discrimination between faces of similar identity (Fig. 14a). These, it seems, are very different tasks, and in part to clarify that our results were particular to movement along the identity axis, we carried out a control experiment using a procedure modified from the previous discrimination experiment. Specifically we measured the minimum % of face 43 (a face that previously generated strong dips for subjects SCD and DEO; Figs. 7 and 8) that had to be added to a pedestal to produce reliable performance on an odd-man-out task. However, now the pedestal was no longer face 43 (at different identity levels) but another face (28) at different identity levels. Thus the subject still had to determine which face was most like face 43, but now the reference faces frequently looked like face 28, and the target was a mixture of 43 and 28. Fig. 14b plots data from this experiment (open symbols, subject SCD) and compares them to results from the last experiment (solid line). Results are unequivocal. Now we observe a steady rise in thresholds with increasing identity level of the pedestal which now serves only to mask the identity of the target; there is no indication of non-linearity beyond a modest saturation of thresholds at the highest level of pedestal identity. Thus we have produced a similar finding to Wilson et al. (2002) – it is harder to spot identity change in faces further from the mean. Combined with our earlier results we can conclude that the discriminability of faces depends not only on the distance they are from the average, *but the direction one pushes them in face space*. Our results indicate that pushing an average along an identity axis (broadly) improves discriminability, whereas Wilson et al.‘s (2002) findings indicate that pushing in directions which transform one face into another are most noticeable when applied to average faces.

It is interesting to consider our findings in the light of evidence indicating categorical processes in face perception. In particular, if one makes a morph continuum going from face A to face B (i.e. faces are mixtures of the identities of A and B in some proportion) and takes pairs of faces differing by 20% in the contribution of each face (e.g. [0.1A + 0.9B, 0.3A + 0.7B] or [0.7A + 0.3B, 0.9A + 0.1B]) then observers are best at categorising the members of faces-pairs as A or B when the pairing straddles a category boundary (e.g. [0.4A + 0.6B,0.6A + 0.4B] assuming that the category boundary is at 0.5A + 0.5B) (Levin & Beale, 2000). This is a highly counter-intuitive result since one might expect best categorisation when one of the faces was clearly either A or B. A reviewer of this paper suggested subjects might be treating the target-face and the average as separate categories, and that optimal discrimination arises at intermediate identity levels arises because we don’t have to push faces very far along the identity axis in order to get them to fall into another distinct category. At present our experiments are unable to separate the predictions of a model based on faces being represented in a continuous space or according to categories; indeed we suggest that non-linearities in a continuous space could be the basis of such categorical effects.

### 6.2 Relationship to neural response

An aim of our experiment was to compare IRFs to results from previous fMRI (Loffler et al., 2005) and electrophysiology (Leopold et al., 2006) studies. Leopold et al. (2006) showed that single neurons in the inferotemporal cortex increase their firing rate near-linearly as a function of increasing identity (up to 160%) in facial stimuli. In addition, Loeffler, et al. (2005) located a region of the inferotemporal cortex which responded more to faces than to other presented objects e.g. houses by presenting faces with varying *geometric distance* from the mean to subjects while measuring fMRI BOLD signal. Under these conditions, BOLD response follows a sigmoidal response to increasing identity that Loffler et al. reason may in part reflect intrinsic properties of the haemodynamic response. These authors conclude that there is likely a monotonic but non-linear relationship of BOLD response to increasing identity. Our inferred underlying response functions for upright faces, exhibit just such a monotonic but non-linear transduction of identity.

Our results indicate that the mapping from identity-level to the dimensions of face space is modestly non-linear, but that this mapping is broadly consistent across faces, being scaled only by the distinctiveness of faces. How might this non-linearity be related to neural responses? We speculate that our findings might be explicable based on the following three assumptions:

(1) Single neurons are broadly tuned to facial identity and exhibit a near-linear dependence of spike rate on identity level (Young & Yamane, 1992).
(2) Face processing uses a sparse population code using the joint contribution of neural sub-populations, composed of elements with properties similar to Assumption 1 (Young & Yamane, 1992).
(3) Discrimination is based on the pooled activity of a set of IT neurons that are prone to multiplicative noise (i.e. becoming more variable as they become more active) (Carandini, 2004; Tolhurst, Movshon, et al., 1981).

The final point is key in proposing that overall activity determines discrimination, and that the more active neurons are, the less reliable their responses become. It is a consequence of such a view that best discrimination occurs with stimuli eliciting low levels of neural activity. Under this hypothesis near-average faces elicit a modest amount of activity from a large number of broadly tuned face sensitive cells (since, by definition, the average face is less different from a neurons preferred face than any other face); this is *dense-low* activity. As the level of identity increases, this activity decreases (as the face stimuli are now decreasingly well matched to the preference of many neurons) leading to correspondingly better discrimination performance (based on the argument that less active neurons produce more reliable signals). As identity level increases beyond the optimal point for discrimination, inappropriately-tuned neurons shut down leaving the sub-population of neurons encoding a particular facial identity to become more and more active and so increasingly dominate activity. An increasingly *sparse-high* pattern of activity will lead to a drop in overall performance (less reliable discrimination) as a result of the increasingly noisy firing of finely tuned neurons at extreme levels of identity. In short we propose that dips arise at the point of optimised, low neural activity, between the extremes of unselective broadly tuned neurons firing at low levels of identity and finely-tuned identity neurons firing very strongly (but unreliably) at extreme levels of identity.

What of the steady increase we observe as we increase the identity level of inverted faces? Above we suggested that this could be due to a reliance on local analysis in inverted faces, which is increasingly suitable at high levels of identity/distortion. We further speculate that this strategy may arise from local components of inverted faces eliciting dense-low activity by weakly stimulating neurons tuned to upright faces. Activity drops with increasing identity as local components becomes more identity-specific and so discrimination improves. We do not observe a subsequent deterioration in performance (as we would for the rising part of a dipper) simply because there are no cells tuned to code the identity of full-identity inverted stimuli, and so no way for the system to switch into a sparse-high state. Instead we simply observe a steady improvement in discrimination as activity falls. Although speculative these are testable hypotheses via examination of the activity of population of neurons in candidate face coding areas in the primate cortex (Tsao, Freiwald, et al., 2006).
